# Mitochondrial diseases in Hong Kong: prevalence, clinical characteristics and genetic landscape

**DOI:** 10.1186/s13023-023-02632-6

**Published:** 2023-03-02

**Authors:** Tsz-sum Wong, Kiran M. Belaramani, Chun-kong Chan, Wing-ki Chan, Wai-lun Larry Chan, Shek-kwan Chang, Sing-ngai Cheung, Ka-yin Cheung, Yuk-fai Cheung, Shuk-ching Josephine Chong, Chi-kwan Jasmine Chow, Hon-yin Brian Chung, Sin-ying Florence Fan, Wai-ming Joshua Fok, Ka-wing Fong, Tsui-hang Sharon Fung, Kwok-fai Hui, Ting-hin Hui, Joannie Hui, Chun-hung Ko, Min-chung Kwan, Mei-kwan Anne Kwok, Sung-shing Jeffrey Kwok, Moon-sing Lai, Yau-on Lam, Ching-wan Lam, Ming-chung Lau, Chun-yiu Eric Law, Wing-cheong Lee, Han-chih Hencher Lee, Chin-nam Lee, Kin-hang Leung, Kit-yan Leung, Siu-hung Li, Tsz-ki Jacky Ling, Kam-tim Timothy Liu, Fai-man Lo, Hiu-tung Lui, Ching-on Luk, Ho-ming Luk, Che-kwan Ma, Karen Ma, Kam-hung Ma, Yuen-ni Mew, Alex Mo, Sui-fun Ng, Wing-kit Grace Poon, Richard Rodenburg, Bun Sheng, Jan Smeitink, Cheuk-ling Charing Szeto, Shuk-mui Tai, Choi-ting Alan Tse, Li-yan Lilian Tsung, Ho-ming June Wong, Wing-yin Winnie Wong, Kwok-kui Wong, Suet-na Sheila Wong, Chun-nei Virginia Wong, Wai-shan Sammy Wong, Chi-kin Felix Wong, Shun-ping Wu, Hiu-fung Jerome Wu, Man-mut Yau, Kin-cheong Eric Yau, Wai-lan Yeung, Hon-ming Jonas Yeung, Kin-keung Edwin Yip, Pui-hong Terence Young, Gao Yuan, Yuet-ping Liz Yuen, Chi-lap Yuen, Cheuk-wing Fung

**Affiliations:** 1 Department of Paediatrics and Adolescent Medicine, Princess Margaret Hospital, Hong Kong, SAR, People’s Republic of China; 2 Department of Paediatrics and Adolescent Medicine, Hong Kong Children’s Hospital, Hong Kong, SAR, People’s Republic of China; 3 Department of Medicine and Geriatrics, United Christian Hospital, Hong Kong, SAR, People’s Republic of China; 4 Department of Medicine, Alice Ho Miu Ling Nethersole Hospital, Hong Kong, SAR, People’s Republic of China; 5 Department of Medicine, Queen Mary Hospital, Hong Kong, SAR, People’s Republic of China; 6 Department of Medicine and Geriatrics, Kwong Wah Hospital, Hong Kong, SAR, People’s Republic of China; 7 Department of Medicine, Queen Elizabeth Hospital, Hong Kong, SAR, People’s Republic of China; 8 Department of Paediatrics, Prince of Wales Hospital, The Chinese University of Hong Kong, Hong Kong, SAR, People’s Republic of China; 9 Department of Paediatrics and Adolescent Medicine, Queen Elizabeth Hospital, Hong Kong, SAR, People’s Republic of China; 10 Department of Paediatrics & Adolescent Medicine, School of Clinical Medicine, LKS Faculty of Medicine, The University of Hong Kong, Hong Kong, SAR, People’s Republic of China; 11 Hong Kong Genome Institute, Hong Kong, SAR, People’s Republic of China; 12 Department of Medicine and Therapeutics, Prince of Wales Hospital, Hong Kong, SAR, People’s Republic of China; 13 Department of Medicine, Yan Chai Hospital, Hong Kong, SAR, People’s Republic of China; 14 Department of Paediatrics and Adolescent Medicine, Kwong Wah Hospital, Hong Kong, SAR, People’s Republic of China; 15 Department of Paediatrics and Adolescent Medicine, Caritas Medical Centre, Hong Kong, SAR, People’s Republic of China; 16 Department of Chemical Pathology, Prince of Wales Hospital, Hong Kong, SAR, People’s Republic of China; 17 Department of Medicine, North District Hospital, Hong Kong, SAR, People’s Republic of China; 18 Department of Pathology, The University of Hong Kong, Hong Kong, SAR, People’s Republic of China; 19 Department of Paediatrics and Adolescent Medicine, United Christian Hospital, Hong Kong, SAR, People’s Republic of China; 20 Department of Chemical Pathology, Queen Mary Hospital, Hong Kong, SAR, People’s Republic of China; 21 Department of Paediatrics and Adolescent Medicine, Pamela Youde Nethersole Eastern Hospital, Hong Kong, SAR, People’s Republic of China; 22 Department of Chemical Pathology, Princess Margaret Hospital, Hong Kong, SAR, People’s Republic of China; 23 Department of Medicine, Pamela Youde Nethersole Eastern Hospital, Hong Kong, SAR, People’s Republic of China; 24 Department of Health, Clinical Genetic Service, Hong Kong, SAR, People’s Republic of China; 25 Department of Medicine, Tseung Kwan O Hospital, Hong Kong, SAR, People’s Republic of China; 26 Clinical Genetics Service Unit, Hong Kong Children’s Hospital, Hong Kong, SAR, People’s Republic of China; 27 Department of Paediatrics and Adolescent Medicine, Alice Ho Miu Ling Nethersole hospital, Hong Kong, SAR, People’s Republic of China; 28 Department of Paediatrics and Adolescent Medicine, Queen Mary Hospital, Hong Kong, SAR, People’s Republic of China; 29 Department of Paediatrics, Radboud Centre for Mitochondrial Medicine, Radboud Institute for Molecular Life Sciences, Radboud University Nijmegen Medicine Centre, Nijmegen, The Netherlands; 30 Department of Medicine and Geriatrics, Princess Margaret Hospital, Hong Kong, SAR, People’s Republic of China; 31 Department of Medicine and Geriatrics, Caritas Medical Centre, Hong Kong, SAR, People’s Republic of China; 32 Department of Pathology, Queen Elizabeth Hospital, Hong Kong, SAR, People’s Republic of China; 33 Department of Paediatrics and Adolescent Medicine, Tseung Kwan O Hospital, Hong Kong, SAR, People’s Republic of China; 34 Department of Medicine and Geriatrics, Ruttonjee and Tang Shiu Kin Hospitals, Hong Kong, SAR, People’s Republic of China; 35 Department of Chemical Pathology, Hong Kong Children’s Hospital, Hong Kong, SAR, People’s Republic of China; 36 Department of Medicine and Geriatrics, Tuen Mun Hospital, Hong Kong, SAR, People’s Republic of China

**Keywords:** Mitochondrial diseases, Prevalence, Hong Kong

## Abstract

**Objective:**

To determine the prevalence of mitochondrial diseases (MD) in Hong Kong (HK) and to evaluate the clinical characteristics and genetic landscape of MD patients in the region.

**Methods:**

This study retrospectively reviewed the phenotypic and molecular characteristics of MD patients from participating public hospitals in HK between January 1985 to October 2020. Molecularly and/or enzymatically confirmed MD cases of any age were recruited via the Clinical Analysis and Reporting System (CDARS) using relevant keywords and/or International Classification of Disease (ICD) codes under the HK Hospital Authority or through the personal recollection of treating clinicians among the investigators.

**Results:**

A total of 119 MD patients were recruited and analyzed in the study. The point prevalence of MD in HK was 1.02 in 100,000 people (95% confidence interval 0.81–1.28 in 100,000). 110 patients had molecularly proven MD and the other nine were diagnosed by OXPHOS enzymology analysis or mitochondrial DNA depletion analysis with unknown molecular basis. Pathogenic variants in the mitochondrial genome (72 patients) were more prevalent than those in the nuclear genome (38 patients) in our cohort. The most commonly involved organ system at disease onset was the neurological system, in which developmental delay, seizures or epilepsy, and stroke-like episodes were the most frequently reported presentations. The mortality rate in our cohort was 37%.

**Conclusion:**

This study is a territory-wide overview of the clinical and genetic characteristics of MD patients in a Chinese population, providing the first available prevalence rate of MD in Hong Kong. The findings of this study aim to facilitate future in-depth evaluation of MD and lay the foundation to establish a local MD registry.

**Supplementary Information:**

The online version contains supplementary material available at 10.1186/s13023-023-02632-6.

## Introduction

Primary mitochondrial diseases (MD) are a group of genetically and phenotypically heterogenous disorders resulting from hampered oxidative phosphorylation system due to defects in the mitochondrial or nuclear genome. Although classically affecting organs with high energy requirement resulting in multisystem involvement, some patients may only present with single system involvement, such as the neurological system [1].

The prevalence of MD in Western countries was reported to be at least 1 in 5000 to 1 in 10,000 [2, 3], but its prevalence in the Chinese population is yet to be reported. Due to the low prevalence of rare diseases like MD, these diseases are often characterized by the gaps of knowledge and expertise among medical practitioners. Yet, these diseases can be debilitating and life-threatening, with detrimental effects on the lives of the patients and their caregivers. Studying the prevalence of rare diseases serves as the first step to define their impact on the population and the socioeconomic costs associated with these diseases. With better understanding of the local disease burden and disease spectrum, registries and targeted surveillance systems could then be developed to raise local awareness and to facilitate early detection of the disease. After more standardized record keeping and registries are in place, clinical trials could then be conducted to develop treatments for these rare diseases, ultimately aiming to improve the care and quality of life of the patients and their families [4, 5].

Unfortunately, potential treatments are only available for a small proportion of MD, including the replacement of coenzyme Q (COQ) in genetic defects affecting COQ biosynthesis and the use of nucleoside precursors in Thymidine Kinase 2 deficiency etc. [6]. Majority of patients with MD remain untreatable [6]. Major reasons to hamper the development of treatment for MD are the extremely heterogeneous clinical phenotypes and molecular etiologies, logistic issues in studying rare diseases, and the incomplete understanding of the pathophysiology of MD. More than 400 genes are now known to cause MD, encompassing several hundreds of orphan diseases [7]. It is therefore nearly impossible to design drugs or gene therapy to treat all these different genetic diseases as a group [8]. A recent Cochrane review suggested that there was no clear evidence to have any effective intervention in MD [9]. The search for a cure for MD has been ongoing in the past decade by different researchers around the world without major success. Various small molecules have been developed and many clinical trials are on-going [10–12].

The primary aim of this study is to provide an updated prevalence of MD in Hong Kong (HK) through collaborations with local public hospitals including both paediatric and adult patients, and to evaluate the clinical and genetic landscape of MD patients.

## Methods

This study was a retrospective review of MD patients in HK. The period of data obtained was from 1st January 1985 to 31st October 2020. Patients were recruited through the Clinical Analysis and Reporting System (CDARS) using relevant keywords and/or International Classification of Diseases (ICD) codes listed in Additional file 1: Table S1. CDARS is a tool developed by the HK Hospital Authority (HA), which manages HK’s public hospitals, to aid users in retrieving clinical information captured by innovative technology systems under the HA. By selecting specific search criteria and filters, relevant patient lists would be generated for the users. Cases could also be recruited through the primary investigators based on their personal recollection of the old cases before the era of CDARS in 1995.

Data from each eligible patient was first obtained by the investigator through the Electronic Patient Record (ePR) system. The ePR system is an electronic system of the HA containing patients’ past medical and nursing documentations, allied health documentations, medication history, operation and procedure records, and investigation results. If the information cannot be retrieved via the ePR system, the investigator had to obtain hard copies of the medical records for data entry.

The inclusion criteria were patients of any age with a confirmed diagnosis of MD, either by respiratory chain enzymology and/or molecular investigations. Patients that were diagnosed clinically without a molecular or functional diagnosis of MD were excluded. Both paediatric and adult units of public hospitals providing primary care for MD patients under the HA were invited to participate. The Institutional Review Board (IRB) numbers of each involved hospital or cluster of hospitals are listed in Additional file 1: Table S2.

Most OXPHOS enzymology analyses in our cohort were performed by the Radboud Centre for Mitochondrial Medicine in the Netherlands. Tissue samples, such as skin and skeletal muscle tissues, were sent to their laboratory to undergo biochemical examination including OXPHOS enzymes and pyruvate dehydrogenase enzyme activities, coenzyme Q10 quantitative analysis, mitochondrial DNA (mtDNA) depletion analysis. A minority of the enzymology analysis done in our cohort were performed in Murdoch Children’s Research Institute of Australia.

Genetic diagnoses were achieved by a variety of genetic techniques depending on clinical context and resource availability. Sanger sequencing of targeted genes was performed mainly in patients with specific clinical phenotypes with known genetic association or in family cascade screening. Next generation sequencing (NGS) gene panel testing and non-targeted NGS such as whole exome sequencing (WES) and whole genome sequencing (WGS) were used for nuclear genome analysis. For the mitochondrial genome, hotspot analysis and targeted gene testing were done using polymerase chain reaction (PCR), restriction fragment length polymorphism, and Sanger sequencing. For selected cases, whole mitochondrial genome sequencing using NGS or molecular analysis for mitochondrial gross deletion using PCR, long-range PCR, and Sanger sequencing, would be performed if clinically indicated. Molecular investigations were provided by laboratories under the HA, the University of Hong Kong, the Chinese University of Hong Kong, Clinical Genetic Service under the Department of Health and the Radboud Centre for Mitochondrial Medicine. Tables 1 and 2 show the distribution of molecular diagnostic methods used for reaching the diagnosis in our cohort.

**Table 1:** Molecular diagnostic methods used in patients with mtDNA pathogenic variants (total 72)

Methods	Number of cases (%)
Targeted mutation analysis	14 (19.4)
Hotspot mutation analysis	28 (38.9)
Long-range PCR for mtDNA deletion	8 (11.1)
Whole mtDNA genome sequencing by NGS	6 (8.3)
Not specified or inadequate documentation	16 (22.2)

*mtDNA* mitochondrial DNA, *NGS* next generation sequencing, *PCR* polymerase chain reaction

**Table 2:** Molecular diagnostic methods used in patients with nDNA pathogenic variants (total 38)

Methods	Number of cases (%)
Targeted mutation analysis	9 (23.7)
Gene panel by NGS	6 (15.8)
Whole exome sequencing by NGS	20 (52.6)
Whole genome sequencing by NGS	3 (7.9)

*nDNA* nuclear DNA, *NGS* next generation sequencing

### Statistical analysis

The demographic variables, clinical characteristics, and genetic characteristics of the patients were mainly analyzed descriptively. The 95% confidence interval for the point prevalence of MD in HK was calculated using the Wilson Score interval. Statistical analyses using Pearson’s Chi-square test and Fisher’s exact test were performed to compare the demographics, clinical, and genetic characteristics of those carrying mtDNA pathogenic variants versus those with nuclear DNA (nDNA) pathogenic variants.

## Results

### Demographics and epidemiology

A total of 119 patients had confirmed MD either by molecular diagnosis and/or enzymatic analysis, including some cases that have been published in the literature [13–23]. The patients’ demographics are summarized in Table 3. Molecular diagnosis was available for 110 patients (92%). The remaining nine out of 119 patients (8%) were diagnosed to have enzyme defects or mtDNA depletion with unknown molecular basis. The time from symptom onset to diagnosis is shown in Table 4.

**Table 3:** Demographics, clinical, and genetic characteristics of Hong Kong Mitochondrial Disease cases (total 119)

Characteristics	Number of patients (% of total)
*Sex*	
Male	65 (54.6)
Female	54 (45.4)
*Ethnicity*	
Chinese	112 (94.1)
Others	7 (5.9)
*Age at onset*	
At birth to < 1 month	14 (11.8)
1 month to < 2 years	35 (29.4)
2 years to < 5 years	9 (7.6)
5 years to < 12 years	21 (17.6)
12 years to < 18 years	12 (10.1)
18 years or older	28 (23.5)
*Clinical syndrome*	
Syndromic	74 (62.2)
Non-syndromic	45 (37.8)
*Family history*	
Positive	51 (42.9)
Patients with mtDNA pathogenic variants	35 (68.6% of 51 cases)
Patients with nDNA pathogenic variants	15 (29.4% of 51 cases)
Unknown molecular basis	1 (2.0% of 51 cases)
Negative	68 (57.1)
*Organ system involvement at presentation*	
Neurological	92 (77.3)
Hearing	14 (11.8)
Gastrointestinal	9 (7.6)
Endocrine	9 (7.6)
Cardiac	6 (5.0)
Renal	4 (3.4)
Ophthalmological	3 (2.5)
Hematological	1 (0.8)
*Molecular genetic information*	
mtDNA pathogenic variants	72 (60.5)
nDNA pathogenic variants	38 (31.9)
Unknown molecular basis	9 (7.6)

*mtDNA* mitochondrial DNA, *nDNA* nuclear DNA

**Table 4:** Time from symptom onset to diagnosis (total 111*)

Time from symptom onset to diagnosis	Number of patients (% of total)
< 2 years	41 (36.9)
2–10 years	36 (32.4)
≥ 10 years	34 (30.6)

Mean number of years from symptom onset to diagnosis was 8.0 years

^*^Total number excluded 5 patients with incomplete data and 3 patients diagnosed by family cascade screening before symptom onset

44 patients had passed away by the end of data collection in October 2020 and thus 75 patients were alive at that point of time. Based on the most recent population census data collected by the HK government in 2016, the total HK population was 7.36 million [24]. The point prevalence was therefore 1.02 in 100,000 people (95% confidence interval: 0.81–1.28 in 100,000), which was significantly lower than the reported Western prevalence of 1 in 5000–10,000 [2, 3].

Most of the cohort belonged to the pediatric age group, while the adult-onset group only accounted for 24% of patients (Table 3). If the two groups are considered separately, the point prevalence of those presenting before 18 years old was 5.24 in 100,000 people (95% confidence interval: 4.04–6.79 in 100,000), a much higher figure than that of the adult-onset group, which was 0.29 in 100,000 people (95% confidence interval: 0.18–0.46 in 100,000).

The incidence of MD between the year 2006 to 2020, expressed in intervals of five years, are listed in Table 5. There was a clear rise in the incidence rate after the year 2010.

**Table 5:** Incidence of Mitochondrial disease in Hong Kong between 2006 and 2020

Years	Incidence (per million inhabitants per year)
2006–2010	0.4 (95% confidence interval: 0.1–1.3)
2011–2015	1.1 (95% confidence interval: 0.5–2.2)
2016–2020	1.4 (95% confidence interval: 0.8–2.6)

### Clinical characteristics

Patients’ clinical characteristics at presentation are shown in Table 3. Due to data incompleteness and inadequate documentation, patients’ clinical manifestations throughout their disease course were not fully analyzed in this study, and thus mainly presentations at disease onset were evaluated. Although MD are well known to be multisystem disorders, 82% of our patients only had single organ system involvement at disease onset. The neurological system (77%) was the most commonly affected organ, followed by the hearing, gastrointestinal, endocrine, and cardiac systems. Of note, none of the patients in our study had hepatic dysfunction as the initial presentation, which was frequently reported in some studies [25, 26]. Specific presentations of individual organ systems at disease onset are listed in Additional file 1: Table S3. Among the neurological presentations, developmental delay with or without regression, seizures, and stroke-like episodes were the most prevalent. Some patients’ phenotypes were complex involving multiple neurological presentations.

To date, MD still lack reliable diagnostic biochemical markers or radiological features [27–29]. Due to the retrospective nature of this study, only plasma lactate and L:P ratio were checked in most of the patients. Although being classical biochemical findings, the absence of hyperlactatemia and elevated L:P ratio do not rule out MD [27–29]. Based on the Nijmegen Mitochondrial Disease Criteria (NMDC), hyperlactatemia was defined as more than two mmol/L detected in more than two separate occasions, while L:P ratio of more than 18 was considered to be elevated [30]. In the 114 patients with plasma lactate checked, 72 patients (63%) had hyperlactatemia, and high L:P ratio was present only in 39 (54%) out of 72 of these patients (data not shown).

**Fig. 1:**
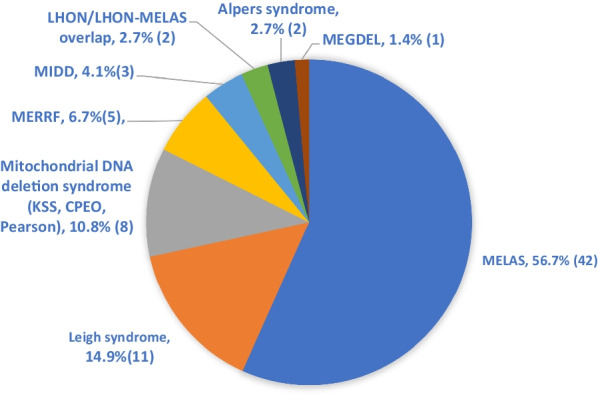
Distribution of Mitochondrial syndromes (total 74). The absolute number of cases are shown in parentheses. Abbreviations: *CPEO* Chronic progressive external ophthalmoplegia, *KSS* Kearns Sayre syndrome, *LHON* Leber hereditary optic neuropathy, *MEGDEL* 3-methylglutaconic aciduria, deafness, encephalopathy, and Leigh-like disease, *MELAS* Mitochondrial Encephalopathy, lactic acidosis, and stroke-like episodes, *MERRF* Myoclonus Epilepsy with ragged red fibers, *MIDD* Maternally Inherited Diabetes and Deafness

MD can be clinically classified into syndromic MD and non-syndromic MD that do not fit into any characteristic phenotypes of MD syndromes reported in the literature [31]. In our study, 74 patients (62%) were classified as syndromic MD (Table 3). Mitochondrial Encephalopathy, Lactic Acidosis, and Stroke-like episode (MELAS), Leigh syndrome, the mtDNA deletion syndromes, and Myoclonus Epilepsy with ragged red fibers (MERRF) were the commonest syndromes identified (Fig. 1). 45 patients (38%) were categorized as non-syndromic MD.

The NMDC scores have been tabulated in our cohort for those 51 patients who had tissue biopsies for histological examination (Additional file 1: Table S4). Possible cases (score two to four), probable cases (score five to seven), and definite cases (score eight to twelve) account for 15.7%, 39.2%, and 45.1%, respectively.

### Family history

51 patients (43%) had documented family history of MD or relatives with typical presentations of MD (Table 3). 35 (69%) of these 51 patients had mtDNA pathogenic variants, 15 (29%) had nDNA pathogenic variants, and one (2%) was diagnosed by OXPHOS with unknown molecular basis. Some relatives with molecularly confirmed MDs were not included in our cohort for various reasons, for instance they did not require management in hospitals in the public sector or they have passed away before the data collection period of this study. If these cases and other oligosymptomatic relatives were included in the calculation of the point prevalence based on the family history data, excluding those that were documented to have succumbed before October 2020, the point prevalence could reach as high as 1.60 in 100,000 people (95% confidence interval: 1.34–1.92 in 100,000).

### Death cases

44 cases have passed away by the end of data collection in October 2020, 52% occurred in adulthood (Additional file 1: Table S5). The mortality rate in our cohort is therefore 37%. Median age of death was 19.3 years old, with a range of 1 month to 60 years old (mean 22.4 years). The age of presentation of the death cases was comparable with the rest of the cohort (P value = 0.142, by Fisher’s exact test, data not shown).

Most patients died of respiratory failure or multi-organ failure. 52% of patients’ deterioration were triggered by an infection, which echoes the findings of other studies examining the causes of death in MD [32]. Notably, ten patients (23%) had sudden unexpected death occurring in either hospital or community setting. Characteristics of these ten patients are shown in Additional file 1: Table S6. Underlying epilepsy or cardiomyopathy were reported to be associated with more severe clinical course and higher risk of death in MD patients [33]. Among these ten patients, seven had epilepsy. Seven patients had echocardiogram done, four of whom had dilated or hypertrophic cardiomyopathy.

### Molecular genetic characteristics

In our study, patients with mtDNA and nDNA pathogenic variants accounted for 61% and 32% of the cases, respectively. The remaining 7% were diagnosed by enzymatic analysis or mtDNA depletion using muscle or skin fibroblasts, with unknown molecular basis. Throughout the diagnostic pathway, a total of 59 patients (49.5%) underwent invasive procedures (Additional file 1: Table S7) such as muscle and skin biopsies. However, molecular study using peripheral blood alone would have reached a genetic diagnosis in 40 (68%) of these 59 patients, which could have potentially spared them from the risks of invasive procedures.

Comparison between patients with mtDNA variants and those with nDNA variants is summarized in Table 6. Although recent advances showed an overlap of age groups of MD resulting from mtDNA and those from nDNA, mtDNA variants classically account for more adult-onset MD, while nDNA variants more frequently result in childhood-onset MD [2]. Similar pattern was observed in our study, showing a statistically significant difference (P < 0.001) in age distribution of the two groups. 26 out of 28 (93%) of the adult-onset cases were caused by mtDNA pathogenic variants. The exceptions were the two cases of autosomal dominant progressive external ophthalmoplegia due to a pathogenic variant in the TWNK gene, presenting at 50 and 72 years old.

**Table 6:** Clinical features of participants with mtDNA pathogenic variants vs nDNA pathogenic variants (total 110)

Characteristics	mtDNA (total 72)	nDNA (total 38)	P value
Age at onset			< 0.001^b^
At birth to < 1 month	1 (1.4%)	7 (18.4%)	
1 month to < 2 years	12 (16.7%)	20 (52.6%)	
2 years to < 5 years	6 (8.3%)	3 (7.9%)	
5 years to < 12 years	16 (22.2%)	5 (13.2%)	
12 years to < 18 years	11 (15.3%)	1 (2.6%)	
18 years or older	26 (36.1%)	2 (5.3%)	
Sex			0.515^a^
Male	37 (51.4%)	22 (57.9%)	
Female	35 (48.6%)	16 (42.1%)	
Organ system involvement at disease onset			
Neurological	55 (76.4%)	33 (86.8%)	0.192^a^
Hearing	10 (13.9%)	2 (5.3%)	0.212^b^
Endocrine	9 (12.5%)	0	0.026^b^
Gastrointestinal	5 (6.9%)	3 (7.9%)	1^b^
Cardiac	1 (1.4%)	4 (10.5%)	0.048^b^
Renal	4 (5.6%)	0	0.296^b^
Eyes	2 (2.8%)	1 (2.6%)	1^b^
Hematology	1 (1.4%)	0	1^b^
Syndromic vs non-syndromic			< 0.001^a^
Syndromic	67 (93.1%)	8 (21.1%)	
Non-syndromic	5 (6.9%)	30 (78.9%)	

*mtDNA* mitochondrial DNA, *nDNA* nuclear DNA

^a^Statistical analysis performed by Pearson’s Chi-Square test

^b^Statistical analysis performed by Fisher’s exact test

Endocrine and cardiac were the organ systems involved at disease onset that showed statistically significant difference between the mtDNA and nDNA groups. Endocrine presentations, including diabetes mellitus and short stature, were only present in the mtDNA group at disease onset. On the other hand, cardiac involvement was more common in the nDNA group.

**Fig. 2:**
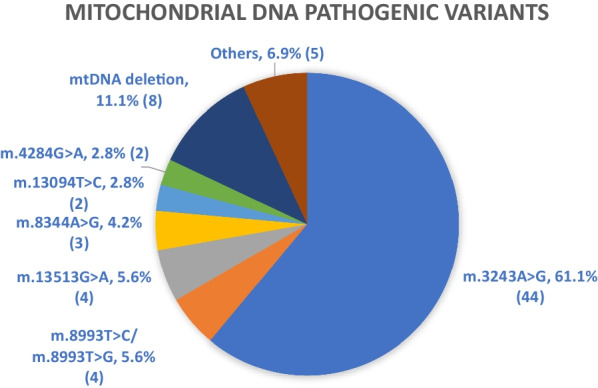
Distribution of mitochondrial DNA pathogenic variants (total 72). The absolute number of cases are shown in parentheses. Others (1.4%/1 patient each): m.10197G>A, m.11778G>A, m.8622delC, m.3946G>A, m.13052G>A. Within these mtDNA pathogenic variants, m.3946G>A, m.13094T>C, and m.13052G>A are not included in the hotspots analyses in several public hospitals. MtDNA deletion also cannot be tested by hotspot analyses. Therefore, total 12 patients (16.7% of total) cannot be identified by hotspot analyses alone

As shown in Fig. 2, among the 72 cases with mtDNA pathogenic variants, 44 (61%) cases had the m.3243A>G variant responsible for most cases of MELAS and Maternally Inherited Diabetes and Deafness (MIDD). Gross mtDNA deletion resulting in Kearns Sayre Syndrome, Pearson Marrow Syndrome, and Chronic Progressive External Ophthalmoplegia, were the second commonest, accounting for 11% of cases with mtDNA pathogenic variants. Other common pathogenic variants included m.13513G>A resulting in Leigh syndrome, m.8993T>C/m.8993T>G causing Neuropathy, ataxia, retinitis pigmentosa syndrome (NARP) and Leigh syndrome, and m.8344A>G causing MERRF. Of note, 83% of these mtDNA variants could be identified by mtDNA panel or hotspot testing available in several public hospitals locally, but the other 17% may be missed if whole mtDNA genome sequencing or deletion and duplication analysis were not performed.

Based on available data, fourteen patients among our cohort were also diagnosed by molecular analysis of the mitochondrial genome using urine samples (Additional file 1: Table S8), a method that is being increasingly recognized for mtDNA analysis due to the high mtDNA load in the epithelial cells of the urinary tract [34, 35]. The m.3243A>G pathogenic variant was identified in ten of these patients, while the other four cases comprised of two cases with the m.4284G>A variant resulting in MERRF, one case of Leigh syndrome due to the m.8993T>G variant, and one case of non-syndromic MD resulting from the m.3946G>A variant. The level of heteroplasmy in blood and urine specimen was available in ten of the cases. In most of these patients (eight out of ten), the level of heteroplasmy was higher in urine than that in blood.

**Fig. 3:**
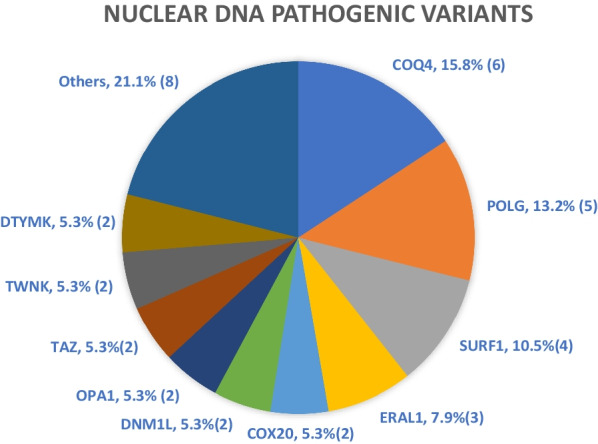
Distribution of nuclear DNA pathogenic variants (total 38). The absolute number of cases are shown in parentheses. Others (2.7%/1 patient each): CHKB, COQ7, GTPBP3, LDHD, NDUFAF5, NDUFA9, NDUFS3, SERAC1

Pathogenic variants on the COQ4 gene were the commonest among the patients with nDNA pathogenic variants (Fig. 3), which was expected as a founder mutation of c.370G>A was demonstrated in the Southern Chinese population [36]. Pathogenic variants in the POLG gene, known to result in mtDNA depletion frequently identified in MD patients in the Western countries, were the second commonest [37]. Of note, the majority (61%) of patients with nDNA pathogenic variants were diagnosed by WES or WGS.

## Discussion

This study is the first overview of the clinical characteristics and genetic landscape of MD patients in the Chinese population. Apart from determining the prevalence of MD in HK, findings from this study serve to improve the awareness of MD among medical practitioners, while also underscoring the challenges that exist in the diagnosis and care of MD patients locally.

### Point prevalence of MD in Hong Kong

The prevalence rate of 1.02 in 100,000 in our study was markedly lower than the reported prevalence of 1 in 5000–10,000 in previous studies [2, 3]. Even if oligosymptomatic relatives and relatives with confirmed MD are included, the prevalence rate was still only 1.60 in 100,000. The prevalence of the pediatric age group was higher at 5.24 in 100,000 people, but was still lower than the quoted figure in the literature, indicating that a proportion of cases may have been missed or under-reported due to various reasons. Firstly, the retrospective nature of the case recruitment method relied mainly on keyword searching using CDARS, which depended heavily on the diagnostic coding entry by the treating clinicians. Such entry was not standardized nor a strict requirement for clinicians, which could lead to inaccurate or missing data. Recruiting cases in the pre-CDARS era using primary investigators’ memory recall was also bound to result in missed cases.

In addition, the participation of investigators in this study was on a voluntary basis and the majority but not all hospitals under HA participated. Moreover, cases exclusively under the care of private practitioners were not recruited. For instance, the m.1555A>G variant known to give rise to non-syndromic hearing impairment and aminoglycoside-induced hearing impairment was absent in our cohort despite being a well-reported variant in the literature [38, 39]. It is postulated that long term management by medical professionals in the public sector may not be strongly indicated for such patients with isolated hearing impairment, and thus led to the under-representation of these patients in our cohort. Similar situations may occur for patients with other milder phenotypes of MD.

Another indication that MD patients may have been under-diagnosed was the prolonged duration between symptoms onset from time of diagnosis. Out of the 111 patients with adequate documentation, the mean number of years from symptom onset to reaching a diagnosis was 8.0 years. In fact, 34 (31%) required more than ten years to reach a diagnosis after symptom onset, where eight of these 34 cases took 30 to 40 years (Table 4). Similar time lag between symptom onset and diagnosis was also reported in other studies, with Pronicka et al. quoting a mean of 7.5 years in their review [40]. When such a large portion of cases went through over a decade of diagnostic odyssey, it can be assumed that some cases of MD were missed or had succumbed before a diagnosis was made. Furthermore, for those with family history of MD, 40% of the patients with mtDNA pathogenic variants had relatives that did not undergo molecular confirmation for MD despite having clinical manifestations of MD. This could be another contributor to an underestimated prevalence rate.

When attempting to identify a molecular diagnosis for suspected MD patients, clinicians must take note of the dual genomes approach in the diagnosis of MD. Clinicians should consider various tests to investigate both the mitochondrial and nuclear genomes in cases of suspected MD, especially for those that do not present as specific mitochondrial syndromes. MtDNA hotspot analysis and deletion/duplication analysis are available in several public hospitals in HK. 60 out of 72 patients (83%) with pathogenic mtDNA variants had mutations included in the hotspot panels. However, the detection of other rarer point mutations and mtDNA deletion require whole mtDNA genome sequencing and/or deletion and duplication analysis. If patients’ mtDNA genetic analysis had terminated at the stage of an unrevealing panel testing, diagnoses of MD resulting from rarer mtDNA pathogenic variants would have been delayed or undiagnosed. The choice of an appropriate tissue for mtDNA analysis due to the heteroplasmy phenomenon also poses extra challenges to the diagnostic evaluation in MD.

Likewise, the majority of patients with nDNA pathogenic variants were diagnosed by WES or WGS (23 out of 38, 61%), in contrast to only 39% identified by a single gene or gene panel approach. In the rapidly expanding landscape of MD-causing nDNA variants, the panel approach is prone to missing newly discovered genes or genes absent from the available panels [40]. In the era in which WES or WGS by NGS is now the recommended choice of MD molecular diagnostics, our study’s findings appear to follow the current trend [40, 41]. Nonetheless, WES has only been more readily available in recent few years, leading to potential under-diagnosis of MD in the past. The limited application of WGS in our cohort is likely another contributing factor to the low prevalence.

Moreover, the lack of local facilities in measuring respiratory chain enzymes activities hindered the diagnosis of MD in HK. Collaboration with overseas centres especially since 2009 successfully diagnosed eight patients having respiratory chain enzyme deficiencies without a positive molecular workup. On the other hand, such collaborative opportunity can be challenging in regional hospitals. Together with the advances in genetics and genomics, there has been a marked increase in MD diagnosed in HK. In our study, among the 113 cases with the date of diagnosis available, 94 (83%) of whom were diagnosed on or after the year 2010 (Data not shown).

The rise in incidence after 2010 was also a clear indication that MD is being increasingly identified with technological advances in diagnostic tools. Between the year 2016 and 2020, the incidence reached 1.40 cases per million people per year. A similar phenomenon was observed in Spain, where a marked rise in incidence to 2.30 cases per million inhabitants per year coincided with the widespread implementation of NGS techniques in the country since 2014 [42].

### Molecular genetic characteristics

Based on our study data, when patients’ manifestations fall into specific mitochondrial syndromes, mtDNA pathogenic variants were more likely to be found (P < 0.001, Table 6). In contrast, abnormalities in the nuclear genome were more common in non-syndromic MD. In our study, adult-onset MD were predominantly caused by mtDNA pathogenic variants while childhood-onset MD, which tend to have more non-specific presentations, were more likely the result of nDNA abnormalities. These observations, which have also been demonstrated in some reviews of MD in the literature, may serve as preliminary guides to the selection of genetic analysis for patients with suspected MD [43, 44]. Yet, these observations need to be interpreted with caution as the m.3243A>G pathogenic mtDNA variant accounts for a large proportion of this cohort (44 out of 119 cases). This may reflect an underlying bias towards screening this pathogenic variant since most HK regional hospitals are able to test it and that it has a strong association with the well-known MELAS phenotype. In addition, clinicians should keep in mind that there are mitochondrial syndromes and phenotypes resulting from nDNA pathogenic variants, such as variants in the POLG and TWNK genes responsible for Alpers syndrome and Autosomal Dominant Progressive External Ophthalmoplegia, respectively [45]. Given the ever-expanding understanding of mitochondrial genomics, an initial unrevealing genetic workup should not discourage clinicians from more in-depth molecular analysis if there is substantial clinical suspicion for MD.

With thorough genetic/genomic analysis, the reliance on invasive procedures such as muscle biopsy is expected to decrease. In our study, 68% of the invasive procedures could have been avoided as genetic analysis of peripheral blood alone would have been sufficient to establish a diagnosis. Prior to the era where NGS technology became readily available for non-targeted molecular diagnostics of MD, “biopsy-first” was still the approach of choice for diagnosing MD. Tissue biopsy may demonstrate essential histological information and can be used for genetic analysis for pathogenic mtDNA variants that may be missed in blood due to heteroplasmy with tissue specificity. For instance, one of our patients with Kearns Sayre syndrome required skeletal muscle samples to detect mtDNA deletion that was not picked up using peripheral blood. However, the drawbacks of tissue biopsy are numerous, including its invasive nature, technical difficulties in specimen handling and transport, and the limited sensitivity and specificity of mitochondrial changes for primary MD [46]. As suggested by recent reviews, a “genetic-first” approach has become the approach of choice, guided by thorough clinical and biochemical assessment [46–48]. If patients’ phenotypes fall into defined MD syndromes, targeted gene analysis or panel approach may be considered. If the phenotypes are non-specific, which is a common scenario in MD, a dual genome non-targeted whole exome or genome sequencing can be performed to look for causative variants.

Clinicians must also be reminded that, if there is substantial clinical suspicion for MD, NGS molecular analysis should not be limited to patients with NMDC scores falling into the probable or definite range. In a study by Witters et al., they reported 12% of cases with nDNA pathogenic variants identified by WES only had an NMDC score of three to four, which fell into the “possible” range [49]. Likewise, in our study, 15.7% of cases with a complete NMDC score available belonged to this range. This phenomenon underscores the possibility of missing MDs if comprehensive molecular evaluation is not applied to cases with lower NMDC scores.

However, even with extensive workup, pathogenic variants may not always be identified. In that situation, clinicians may need to reconsider alternative diagnoses, consider further investigations such as tissue biopsies and urine analysis if clinically indicated, and/or periodically review the genetic findings and the literature for possible novel candidate variants that may explain the clinical presentations. Advanced technologies such as transcriptomics and proteomics, which are being increasingly applied in combination with WGS and WES in the diagnosis of MD, may bring additional value in the workup of undiagnosed MD [50–52].

### Limitations

As mentioned, this study was limited by its retrospective nature, case recruitment method, and the limited application of more advanced diagnostic tools in HK until the recent decade. In addition, despite providing the first wide-ranging overview of MD patients in HK, in-depth analysis of specific aspects of MD was not performed. For instance, comprehensive analysis of various MD syndromes was difficult due to the limited number of cases. Thorough assessment of the systems involvement throughout patients’ disease course was impractical owing to inadequate documentations and non-standardized complications surveillance. The treatment effectiveness of MD was also not evaluated for due to similar reasons. Given these limitations, prospective and standardized natural history studies should be carried out in the future to better understand the clinical manifestations and genetic landscape of MD in HK.

## Conclusion

This study was the first territory-wide review of MD in HK and it established a prevalence rate of 1.02 in 100,000 of MD in the region. With increased availability of more comprehensive diagnostic tools such as WES, this figure is expected to rise. Although limited by its retrospective nature and the lack of in-depth analysis, it provided a wide-ranging evaluation of the clinical and genetic characteristics of MD patients in HK. The findings of this study will facilitate future comprehensive evaluation of MD and lay the foundation to establish a MD registry in HK.

### Supplementary Information

**Additional file 1. Supplementary table 1**. Keywords and/or ICD codes used for recruiting patients through CDARS.** Supplementary table 2**. Institutional Review Board (IRB) numbers of involved hospitals in Hong Kong.** Supplementary table 3**. Clinical presentations by system and age distribution of patients at disease onset.** Supplementary table 4**. NMDC score for cases with histological examination done on biopsy (total 51).** Supplementary table 5**. Characteristics of death cases (total 44).** Supplementary table 6**. Detail of cases with sudden death (total 10).** Supplementary table 7**. Types of tissue samples obtained by invasive procedures (total 59 patients) and the examination performedCharacteristics.

## Data Availability

All available data generated during this study are included in this published article and its additional files.
