# Supplementary material for: Mitochondrial diseases in Hong Kong: prevalence, clinical characteristics and genetic landscape
Source: Orphanet J Rare Dis. 2023 Mar 2;18:43. doi: 10.1186/s13023-023-02632-6 (PMC9979401; doi:10.1186/s13023-023-02632-6)
Supplement: Supplementary file 1 — Additional file 1. Supplementary table 1. Keywords and/or ICD codes used for recruiting patients through CDARS. Supplementary table 2. Institutional Review Board (IRB) numbers of involved hospitals in Hong Kong. Supplementary table 3. Clinical presentations by system and age distribution of patients at disease onset. Supplementary table 4. NMDC score for cases with histological examination done on biopsy (total 51). Supplementary table 5. Characteristics of death cases (total 44). Supplementary table 6. Detail of cases with sudden death (total 10). Supplementary table 7. Types of tissue samples obtained by invasive procedures (total 59 patients) and the examination performedCharacteristics. [file 13023_2023_2632_MOESM1_ESM.docx]

**Supplementary table 1:** **Keywords and/or ICD codes used for recruiting patients through CDARS**

| **CDARS code** | **Keywords/ICD code** |
| --- | --- |
| 277.8 | Pearson syndrome, Kearns Sayre syndrome, Mitochondrial Complex I deficiency, Mitochondrial complex IV deficiency |
| 330.4 | Mitochondrial cytopathy, Myoclonus epilepsy and ragged red fiber, Juvenile Myopathy Encephalopathy Lactic acidosis and Stroke, Mitochondrial myoencephalopathy |
| 330.8 | Leigh’s disease, Alpers-Huttenlocher syndrome |
| 359.8 | Mitochondrial disease, Mitochondrial cytopathy, Mitochondrial lipid glycogen storage myopathy |
| 377.16 | Leber’s optic atrophy |
| 378.72 | Progressive external ophthalmoplegia |
| 277.9 | Inborn error of metabolism^*^ |
| 330.9 | Neurodegenerative disease^*^ |

*^*^ The investigator had to be stringent to ensure the final diagnosis of the patient was MD.*

*Abbreviation: CDARS = Clinical Analysis and Reporting System; ICD = International Classification of Diseases*

**Supplementary table 2: Institutional Review Board (IRB) numbers of involved hospitals in Hong Kong**

| **Hospital** | **IRB number** |
| --- | --- |
| Alice Ho Miu Ling Nethersole Hospital | 2019.018 |
| Hong Kong Children’s Hospital | HKCH-REC-2019-030 |
| Kowloon West Cluster (Princess Margaret Hospital, Yan Chai Hospital, and Caritas Medical Centre) | 127-06 |
| Kwong Wah Hospital | REC(KC/KE)-19-0011/ER-2 |
| North District Hospital | 2018.311 |
| Prince of Wales Hospital | 2019.464 |
| Pamela Youde Nethersole Eastern Hospital | HKECREC-2018-071 |
| Queen Elizabeth Hospital | KC/KE-18-0206/ER-2 |
| Queen Mary Hospital | UW 18-130 |
| Ruttonjee and Tang Shiu Kin Hospitals | HKECREC-2018-058 |
| Tseung Kwan O Hospital | REC(KC/KE)-18-0257/ER-4 |
| United Christian Hospital | REC/KE)-19-0012/ER-2 |

**Supplementary table 3: Clinical presentations by system and age distribution of patients at disease onset**

| **Organ systems involvement (total number of patients)** | **Clinical presentations** | **Number of patients (% of total)** | **Median age (years)** | **Age range (years)** |
| --- | --- | --- | --- | --- |
| Neurological  (92) | Developmental delay or regression | 37 (40.2) | 0.5 | 0-8 |
|  | Epilepsy/seizures | 35 (38.0) | 7.2 | 0-59 |
|  | Stroke-like episodes | 19 (20.7) | 12.2 | 6-55 |
|  | Encephalopathy | 10 (10.9) | 9.6 | 0-34 |
|  | Hypotonia | 9 (9.8) | 0.5 | 0-3 |
|  | Diplopia/ophthalmoplegia | 6 (6.5) | 22 | 10-50 |
|  | Ptosis | 6 (6.5) | 33 | 14-72 |
|  | Extra-pyramidal involvement | 5 (5.4) | 1 | 0.08-8 |
|  | Limb weakness | 4 (4.3) | 5.5 | 1.5-30 |
|  | Headache | 3 (3.3) | 10.25 | 10-11.5 |
|  | Spasticity | 3 (3.3) | 1 | 0.25-3 |
|  | Ataxia | 2 (2.2) | 4.5 | 1-8 |
| Hearing (14) | Hearing impairment ^a,b^ | 14 (100) | 18.5 | 0-51 |
| Gastrointestinal (9) | Failure to thrive | 8 (88.9) | 1.35 | 0.25-10 |
|  | Bowel pseudo-obstruction | 1 (11.1) | 0 ^c^ | 0 ^c^ |
| Endocrine (9) | Diabetes Mellitus | 6 (66.7) | 35.5 | 20-50 |
|  | Short stature | 3 (33.3) | 6 | 2-14 |
| Cardiac (6) | Hypertrophic cardiomyopathy | 2 (33.3) | 0 ^c^ | 0 ^c^ |
|  | Dilated cardiomyopathy | 2 (33.3) | 0.21 | 0-0.42 |
|  | Heart block | 1 (16.7) | 6 | 6 |
|  | Wolff Parkinson White syndrome | 1 (16.7) | 14 | 14 |
| Renal (4) | Fanconi syndrome | 2 (50) | 1.75 | 1.5-2 |
|  | Focal segmental glomerulosclerosis | 1 (25) | 20 | 20 |
|  | Nephrotic range proteinuria (renal biopsy: chronic changes only) | 1 (25) | 30 | 30 |
| Ophthalmological (3) | Optic atrophy | 3 (100) | 14 | 4-15 |
| Hematological (1) | Severe anemia | 1 (100) | 0.8 | 0.8 |

*^a^ Three patients were picked up by newborn hearing screening.*

*^b^ Type of hearing loss: Sensorineural in ten patients, incomplete documentation in four patients, and none was documented as conductive hearing loss.*

*^c^ Onset at newborn period.*

**Supplementary table 4: NMDC score for cases with histological examination done on biopsy (total 51)**

| **NMDC score** | **nDNA (16)** | **mtDNA (27)** | **Unknown molecular basis (8)** | **Sum (51)** |
| --- | --- | --- | --- | --- |
| 2 | 0 | 1 | 0 | 1 |
| 3 | 1 | 2 | 0 | 3 |
| 4 | 0 | 3 | 1 | 4 |
| 5 | 1 | 3 | 0 | 4 |
| 6 | 4 | 3 | 3 | 10 |
| 7 | 3 | 3 | 0 | 6 |
| 8 | 4 | 5 | 3 | 12 |
| 9 | 1 | 4 | 1 | 6 |
| 10 | 2 | 2 | 0 | 4 |
| 11 | 0 | 1 | 0 | 1 |

*Abbreviations: mtDNA = mitochondrial DNA; nDNA = nuclear DNA; NMDC =Nijmegen Mitochondrial Disease Criteria*

*Possible (2-4 points): 15.7%; Probable (5-7 points): 39.2%; Definite (8-12 points): 45.1%*

**Supplementary table 5: Characteristics of death cases (total 44)**

| **Characteristics** | | **Frequency (%)** |
| --- | --- | --- |
| Sex | Male | 26 (59.1) |
|  | Female | 18 (40.9) |
| Age at death | At birth to <1 month | 0 |
|  | 1 month to < 2 years | 6 (13.6) |
|  | 2 years to <5 years | 4 (9.1) |
|  | 5 years to <12 years | 6 (13.6) |
|  | 12 years to <18 years | 5 (11.4) |
|  | 18 years or older | 23 (52.3) |
| Age at presentation | At birth to <1 month | 7 (15.9) |
|  | 1 month to < 2 years | 7 (15.9) |
|  | 2 years to <5 years | 4 (9.1) |
|  | 5 years to <12 years | 11 (25) |
|  | 12 years to <18 years | 5 (11.4) |
|  | 18 years or older | 10 (22.7) |
| Median age of death | 19.3 years | |
| Mean age of death | 22.4 years | |
| Causes of death | Respiratory failure | 13 (29.5) |
|  | Multi-organ failure | 7 (15.9) |
|  | Septic shock | 5 (11.4) |
|  | Cardiac causes (Heart failure, heart block) | 2 (4.5) |
|  | Status epilepticus | 2 (4.5) |
|  | Sudden arrest | 10 (22.7) |
|  | Incomplete data | 5 (11.4) |
| Triggers of deterioration | Infection | 23 (52.3) |
|  | Elective extubation^a^ | 2 (4.5) |
|  | Unknown trigger or disease progression | 18 (40.9) |
| Molecular diagnosis | mtDNA pathogenic variants | 33 (75.0) |
|  | nDNA pathogenic variants | 8 (18.2) |
|  | Unknown molecular basis | 3 (6.8) |

*Abbreviations: mtDNA = mitochondrial DNA; nDNA = nuclear DNA*

*^a^ One case of Primary Coenzyme Q10 Deficiency type 7 and one case of Leigh syndrome. Due to severe respiratory failure and epileptic encephalopathy, management took a palliative approach after discussion with the parents. Patients were extubated electively and succumbed shortly after.*

**Supplementary table 6: Detail of cases with sudden death (total 10)**

|  | **Functional analysis/ molecular diagnosis** | **Syndrome** | **Age of death (years)/ Sex** | **Death events** | **Neurological presentations** | **Cardiac presentations** | **Other features** |
| --- | --- | --- | --- | --- | --- | --- | --- |
| 1 | m.3243A>G | MELAS | 49/M | Arrest in old age home, CPR by ambulance crew | Stroke-like episodes, Seizures on AED  Intellectual disability | Echocardiogram not done, ECG: LVH by voltage | Bowel pseudo-obstruction, SNHL |
| 2 | m.3243A>G | None | 37/M | Arrest at home, CPR by ambulance crew | Nil | Hypertrophic cardiomyopathy | Myopathy |
| 3 | m.3243A>G | MELAS | 58/F | Arrest at home CPR by ambulance crew | Stroke-like episodes, refractory epilepsy on multiple AED | Echocardiogram not done | Hearing impairment |
| 4 | m.3243A>G | MELAS | 15/F | Arrest in rehab hospital, DNACPR | Stroke-like episodes, Epilepsy well-controlled with AED | Dilated cardiomyopathy | DM on insulin, nephrotic syndrome |
| 5 | m.3243A>G | MIDD | 54/M | Arrest in ward, CPR by hospital staff | Nil | Hypertrophic cardiomyopathy | DM on insulin, hypogonadism, SNHL |
| 6 | m.8993T>G | MILS | 0.4/F | Arrest in acute hospital, repeated CPR and organ support | Hypotonia, global delay, no seizures | Hypertrophic cardiomyopathy |  |
| 7 | m.4284G>A | MERRF | 34/M | Arrest at home, CPR by family | Refractory myoclonic epilepsy on multiple AED + vagal nerve stimulator | Echocardiogram not done | Non-insulin dependent DM |
| 8 | SERAC1  (Compound heterozygous:  c.202C>T/  c.1598C>G) | MEGDEL | 8/F | Arrest in rehab hospital, CPR by hospital staff | Epilepsy with good seizure control on Levetiracetam, GDD, choreoathetosis, recurrent stroke | Echocardiogram normal | SNHL, visual impairment |
| 9 | GTPBP3 (Compound heterozygous:  c.152T>A, c.872A>G) | None | 5/M | Arrest in rehab hospital, CPR by hospital staff | Intractable epilepsy on ketogenic diet, GDD, dyskinesia | Echocardiogram yearly normal |  |
| 10 | Complex I and IV deficiency | None | 3/M | Found no signs of life at home by father, CPR by father | Myoclonic seizures, generalized pyramidal and extrapyramidal tract signs, oromotor dysfunction | Echocardiogram normal | Bilateral cataracts, SNHL |

*Abbreviations: AED = Anti-epileptic drugs; CPR = Cardiopulmonary Resuscitation; DM = Diabetes Mellitus; DNACPR = Do-Not-Attempt Cardiopulmonary Resuscitation; ECG = Electrocardiogram; GDD = Global Developmental Delay; MEGDEL = 3-Dethylglutaconic Aciduria, Deafness, Encephalopathy, and Leigh-like disease; MELAS = Mitochondrial Encephalopathy, Lactic Acidosis, and Stroke-like episodes; MERRF = Myoclonus Epilepsy with Ragged Red Fibers; MIDD = Maternally Inherited Diabetes and Deafness; MILS = Maternally Inherited Leigh Syndrome; Rehab = Rehabilitation; SNHL = sensorineural hearing loss.*

**Supplementary table 7: Types of tissue samples obtained by invasive procedures (total 59 patients) and the examination performed**

| **Characteristics** | | **Number of patients (% of total)** |
| --- | --- | --- |
| Tissues samples obtained | Muscle only | 32 (54.2) |
|  | Skin only | 5 (8.5) |
|  | Renal tissue only ^a^ | 1 (1.7) |
|  | Myocardial tissue only ^b^ | 1 (1.7) |
|  | Muscle and skin | 16 (27.1) |
|  | Muscle and kidney ^c, d^ | 2 (3.4) |
|  | Muscle and liver ^e^ | 1 (1.7) |
|  | Muscle, skin and liver ^f^ | 1 (1.7) |
| Examination performed using the tissue samples | Histological only | 24 (40.7) |
|  | OXPHOS only | 5 (8.5) |
|  | Molecular only | 1 (1.7) |
|  | OXPHOS and molecular | 1 (1.7) |
|  | Histological and molecular | 7 (11.9) |
|  | Histological and OXPHOS | 6 (10.2) |
|  | Histological, OXPHOS, and molecular | 15 (25.4) |
| OXPHOS enzymatic defects (done on total 27 patients) | Complex I | 6 (22.2) |
|  | Complex II and III | 3 (11.1) |
|  | Complex IV | 8 (29.6) |
|  | Complex I and IV | 5 (18.5) |
|  | Complex I, III, and IV | 1 (3.7) |
|  | Complex II and III, coenzyme Q | 1 (3.7) |
|  | Normal | 3 (11.1) |

1. *Case of Kearns Sayre syndrome with Fanconi syndrome, histology showed abnormal mitochondria.*
2. *Case of dilated cardiomyopathy. Endomyocardial biopsy taken but was unsatisfactory for evaluation due to poor yield. Patient was diagnosed later by molecular testing in peripheral blood as TAZ-related cardiomyopathy.*
3. *Case of non-syndromic mitochondrial disease with nephrotic range proteinuria; renal biopsy showed chronic changes only. Molecular testing of muscle detected a pathogenic variant of m.13513G>A.*
4. *Case of non-syndromic mitochondrial disease with Fanconi syndrome; renal biopsy showed focal segmental glomerulosclerosis. Molecular testing of muscle and peripheral blood detected a likely pathogenic variant of m.8622delC.*
5. *Case of MEGDEL with negative yield of histological examination in muscle and liver. Patient was diagnosed by whole exome sequencing with peripheral blood, detecting compound heterozygous pathogenic variants of SERAC1.*
6. *Case of non-syndromic complex IV deficiency with unknown molecular basis. OXPHOS enzymology analysis of liver tissue showed mild complex IV deficiency.*

**Supplementary table 8: Detail of cases with mitochondrial genome analysis using urine sample (total 14)**

| **Molecular diagnosis** | **Syndromic diagnosis** | **Level of heteroplasmy in urine** | **Level of heteroplasmy in blood** | **Molecular analysis of other tissues** | **Main features** |
| --- | --- | --- | --- | --- | --- |
| m.3243A>G | MELAS | 34% | 12% | None | DM, cardiomyopathy, myopathy, SNHL, cerebellar atrophy, lactic acidosis |
| m.3243A>G | MELAS | 97% | 67% | None | GDD, dystonia, myopathy, SNHL, FTT, lactic acidosis |
| m.3243A>G | MELAS | Detected^a^ | Not detected | None | Epilepsy, myopathy |
| m.3243A>G | MELAS | Detected^a^ | Detected^a^ | None | Stroke-like episodes, encephalopathy, epilepsy, myopathy,  dilated cardiomyopathy, lactic acidosis |
| m.3243A>G | MELAS | 58% | 17% | None | Encephalopathy, CI, DM |
| m.3243A>G | MELAS | Detected^a^ | Detected^a^ | None | Stroke-like episodes, epilepsy, pigmentary retinopathy, SNHL |
| m.3243A>G | MELAS | 94% | 50% | None | Stroke-like episodes, myopathy, CI, lactic acidosis |
| m.3243A>G | MELAS | 95% | 61% | None | Stroke-like episodes, migraine headache, CI, exercise intolerance, lactic acidosis |
| m.3243A>G | MELAS | Detected^a^ | Detected^a^ | None | Stroke-like episodes, encephalopathy, CI |
| m.3243A>G | None | 52% | 41% | None | Myopathy, lactic acidosis |
| m.4284G>A | MERRF | 83% | 65% | Skeletal muscle: Apparently homoplasmic | Myoclonic epilepsy, DM, CI, SNHL, lactic acidosis |
| m.4284G>A | MERRF | 40% | 60% | None | Myoclonic seizures |
| m.8993T>G | Leigh syndrome | Homoplasmic | Homoplasmic | None | Epilepsy, encephalopathy, central apnea, renal tubular acidosis, SNHL |
| m.3946G>A | None | 89% | 70% | None | GDD, Wolff Parkinson White syndrome, lactic acidosis |

*Abbreviations: CI = Cognitive impairment; CKD = Chronic kidney disease; DM = Diabetes Mellitus; FTT = Failure to thrive; GDD = Global developmental delay; LA = Lactic Acidosis; MELAS = Mitochondrial Encephalopathy, Lactic Acidosis, and Stroke-like episodes; MERRF = Myoclonus Epilepsy with Ragged Red Fibers; RFLP = Restriction Fragment Length Polymorphism; SNHL = Sensorineural hearing loss*

*^a^ Level of heteroplasmy not mentioned in available reports.*
